# Seroprevalence of neuronal antibodies in diseases mimicking autoimmune encephalitis

**DOI:** 10.1038/s41598-024-55995-6

**Published:** 2024-03-04

**Authors:** Mantas Vaisvilas, David Petrosian, Loreta Bagdonaite, Vera Taluntiene, Viktorija Kralikiene, Neringa Daugelaviciene, Urte Neniskyte, Gintaras Kaubrys, Natasa Giedraitiene

**Affiliations:** 1 Clinic of Neurology and Neurosurgery, Faculty of Medicine, Institute of Clinical Medicine, Vilnius University, Vilnius, Lithuania; 2 Faculty of Medicine, Vilnius University, Vilnius, Lithuania; 3 Department of Laboratory Medicine, Vilnius University Hospital Santaros Klinikos, Vilnius, Lithuania; 4 Institute of Biosciences, Life Sciences Center, Vilnius University, Vilnius, Lithuania; 5 VU LSC-EMBL Partnership for Genome Editing Technologies, Life Sciences Center, Vilnius University, Vilnius, Lithuania; 6 Department of Neurology, Vilnius University Hospital Santaros Klinikos, Santariskiu str. 2, 08661 Vilnius, Lithuania

**Keywords:** Neuroimmunology, Multiple sclerosis

## Abstract

Detection of neuronal antibodies for autoimmune encephalitis and paraneoplastic neurological syndromes relies on commercially available cell-based assays and lineblots. However, lineblots may reveal the presence of neuronal antibodies in patients with various non-autoimmune etiologies. Herein we describe patients with non-autoimmune etiologies (cohort B) and detectable neuronal antibodies and compare them to definite cases of autoimmune encephalitis (cohort A) for differences in clinical data. All patients positive for at least one neuronal antibody were retrospectively evaluated for autoimmune encephalitis and/or paraneoplastic neurological syndrome between 2016 and 2022. 39 cases in cohort B and 23 in cohort A were identified. In cohort B, most common diagnoses were neurodegenerative disorders in 9/39 (23.1%), brain tumors in 6/39 (15.4%) while most common detected antibodies were anti–titin (N10), anti-recoverin (N11), anti-Yo (N8) and all were detected in serum only. Differential aspects between cohort A and B were CSF pleocytosis (14/23 (60.8%) vs 11/35 (31.4%), p = 0.042, respectively), MRI features suggestive of encephalitis (6/23 (26.1%) vs 0 (0%), p = 0.002, respectively) and epilepsy restricted to temporal lobes (14/23 (60.9%) vs 2/30 (6.7%), p = 0.0003, respectively). A large proportion of lineblot results were non-specific when only serum was tested and were frequently found in non-autoimmune neurological conditions.

## Introduction

Autoimmune encephalitis (AE) and paraneoplastic neurological syndromes (PNS) are rare autoimmune nervous system disorders with distinct clinical presentations, most commonly limbic encephalitis, rapidly progressive cerebellar syndrome, etc^1^. Therefore, diagnosis of these disorders relies on clinical criteria reflecting dysfunction mainly of the limbic system with evidence of neuroinflammation otherwise not explained by any other causes^2,3^. In parallel, detection of neuronal antibodies in AE and PNS ascertains the diagnosis of these conditions in typical presentations, makes diagnosis possible for cases where non-limbic involvement is prominent and guides cancer screening in cases of PNS^4^. In current clinical practice, commercial assays allowing detection of both surface and intracellular neuronal antibodies are commonly applied.

On one hand, commercial assays are a practical and time-effective approach allowing simultaneous detection of numerous antibodies using a single assay. On the other hand, regarding the detection of intracellular antibodies, a high proportion of false positive antibody results not confirmable with alternative diagnostic assays and alternative explanations of neurological symptoms have been reported^5–7^. Caveats of commercial assays may be ameliorated with additional diagnostic modalities available in specialized research laboratories. However, current number of research centers with advanced immunological testing is low, while the demand for neuronal antibody testing is high and growing rapidly. Consequently, most clinical centers rely not only on commercial assays alone, but importantly, results from serum without additional cerebrospinal fluid (CSF) testing due to lower sensitivity of CSF samples^8^.

This leads to the high rate of misdiagnosis of various primary neurological and psychiatric disorders for AE or PNS^9–11^. This is a growing concern because unnecessary long-term immunosuppression is incorrectly applied offering false aspirations for patients and their caregivers in hope of a treatable neurological disorder, is associated with numerous complications of long-term immunosuppression and, importantly, misguides clinicians and researchers, when cases with non-specific antibody results are presented as “expanding phenotypes” of antibody-positive neurological disorders in the scientific literature. Therefore, it is essential to highlight clinical scenarios of neuronal antibody positivity in AE or PNS mimics. However, current data regarding the mimics is scarce.

Therefore, we aimed to characterize cases of detectable neuronal antibodies with commercial assays and alternative diagnoses to AE and PNS. To highlight the differences between definite AE/PNS cases and their mimics, we compared clinical data between the two groups from our center.

### Ethical considerations

This study was approved on 2023 04 18 by the Lithuanian Bioethics Committee. Approval number No. L-23–02/2. All methods were performed in accordance with the relevant guidelines and regulations. Additionally, for publication of identifying images in an open-access publication, informed consent was obtained from all subjects or their legal guardians. For experiments with human samples/tissues, we confirm that informed consent was obtained from all subjects and/or their legal guardian(s).

## RESULTS

### General cohort description

Between 2016 and 2022, 1004 samples were tested and 74 (7.3%) were positive for at least one neuronal antibody. Excluding antibody-associated demyelinating diseases (11/70, 15.7%), 45/63 (71.4%) of samples were positive for intracellular and 18/63 (28.6%) for neuronal surface antibodies. 11/63 (17.4%) samples were positive for more than one antibody. Most detected antibodies against intracellular antigens were anti-Yo (14/45, 31.1%), anti- recoverin (11/45, 24.4%) and anti-titin (10/45, 22.2%) overlapping with either anti-Yo or anti-recoverin in 6/10 (60%) of cases. Most detected neuronal surface antibodies were anti-NMDAR (5/18, 27.8%) and anti-voltage gated potassium channel complex (VGKC) (anti-CASPR2 3/18, 16.6% or anti-LGI-1 4/18, 22.2%).

In 39/63 (61.9%) of cases, antibody results were considered non-specific. Of these 39 cases, 35 (89.7%) had alternative diagnoses and 4 (10.2%) had autoimmune limbic encephalitis (discussed separately). However, in the 4 cases the relationship between limbic encephalitis and detected antibodies was considered unlikely therefore these patients were also included in the analysis and are discussed separately.

### Study cohort (cohort B)

39 patients were included and 23 (59.1%) were female. Median age of the entire cohort was 65 (IQR 53–72) years. Major complaints were cognitive decline in 15/39 (38.4%) followed by movement disorders including cerebellar and extrapyramidal involvement in 8/39 (20.8%) (Table 1). In 34/39 (87.1%) of cases, presenting constellation of symptoms were not compatible with possible AE criteria and were progressive with a median of 6 months (IQR 1–22) from symptom onset. However, 5/39 (12.9%) cases fulfilled the criteria of possible AE and 3/5 had definite autoimmune limbic encephalitis (discussed below). Most common established diagnoses were neurodegenerative disorders in 9/39 (23.1%), symptoms associated with direct effects or primary or metastatic brain tumors in 6/39 (15.4%), neuroinfectious in 4/39 (10.2%), primary psychiatric in 2/39 (5.1%) and miscellaneous causes in 10/39 (25.6%) of cases, most commonly – isolated cryptogenic epilepsy, vestibular dysfunction-associated imbalance and muscular disorders.

**Table 1:** Clinicodemographic data of Cohort A.

	Cohort A N = 39
Age, median (IQR)	65 (53–72)
Female, N (%)	23 (59.1)
Detected antibody	
Intracellular	
Anti-Hu, N (%)	1 (2.6)
Anti-Ri, N (%)	0 (0)
Anti-Ma1/2, N (%)	1 (2.6)
Anti-amphiphysin, N (%)	7 (17.9)
Anti-CV2/CRMP5, N (%)	1 (2.6)
Anti-Yo, N (%)	8 (8.1)
Anti-Ta, N (%)	0 (0)
Anti-titin, N (%)	10(25.6)
Anti-recoverin, N (%)	11(28.2)
Anti-SOX-1	7 (17.9)
Neuronal surface	
Anti-NMDAR, N (%)	3 (7.6)
Time from symptom onset to antibody result, median months (IQR)	6 (1–22)
Main clinical syndrome during antibody testing	
Cognitive decline, N (%)	15 (38.4)
Movement disorder, N (%)	4 (8.9)
Cerebellar syndrome, N (%)	4 (8.9)
Isolated seizures, N (%)	3 (6.7)
Cortical deficit, N (%)	3 (6.7)
Psychiatric, N (%)	2 (4.4)
Other manifestations N (%)	8 (17.8)
MRI features (N30)	
Normal, N (%)	8 (26.7)
Cancer-specific, N (%)	5 (16.7)
Neurodegeneration specific, N (%)	9 (30)
Cerebrovascular, N (%)	2 (6.7)
Neuroinfectious, N (%)	3 (10)
Limbic encephalitis, N (%)	3 (10)
CSF features	
Cell count, median (IQR)	2 (1–5)
Protein (g/L), median (IQR)	0.463 (0.286–0.622)
Oligoclonal bands (N7), N (%)	0 (0)
EEG features (N30)	
Normal, N (%)	2 (4.4)
Non-specific, N (%)	16 (35.6)
Focal slowing, N (%)	2 (4.4.)
Generalized slowing, N (%)	6 (13.3)
Focal epilepsy, N (%)	4 (8.9)
Generalized epilepsy, N (%)	0 (0)
Final diagnosis	
Neurodegenerative, N (%)^§^	9 (23.1%)
Cerebrovascular, N (%)^§§§^	2 (4.4)
Infectious, N (%)^§§^	4 (8.9)
Cancer, N (%)	6 (15.4)
Primary psychiatric, N (%)	2 (4.4)
Metabolic, N (%)*	2 (4.4)
Limbic encephalitis, N (%)	4 (8.9)
Other, N (%)**	10 (25.6)
PNS-care score, median (IQR)	3 (1–3)
Last known follow-up (> 12 months), median (IQR)	27 (17–33)
Fulfills possible AE criteria , N (%)	5 (12.8)
Part 1 of the criteria N (%)	5 (12.8)
Part 2 of the criteria N (%)	7 (17.9)

Metastatic solid organ or primary central nervous system glial or hematological malignancies and central nervous system leukemia; Long established paranoid schizophrenia* AE* autoimmune encephalitis, *CRMP5* collapsin response-mediator protein-5, *CSF* cerebral spinal fluid, *DNER* Delta/Notch-like epidermal growth factor-related receptor, *EEG* electroencephalography, *MRI* magnetic resonance imaging, *NMDAR* N-methyl-D-aspartate- receptor, *PCA-1* Purkinje cell cytoplasmic antibody-1, *PNS* paraneoplastic neurological syndromes.

^**§**^Includes Alzheimer’s disease, dementia with Lewy bodies, Multisystem atrophy, Parkinson disease, olivopontocerebellar degeneration, inherited leukodystrophy and corticobasal syndrome, ^**§§**^Includes viral encephalitis and Creutzfeldt-Jacob disease, ^§§§^Acute cerebrovascular events.

*Includes Wernicke encephalopathy, **Includes peripheral vestibular dysfunction, peripheral neuropathy, myopathy/myositis, cryptogenic and poststroke epilepsy, end-stage cancer induced cachexia, orthostatic intolerance, transient global amnesi.

### Paraclinical findings

Supportive findings for alternative diagnoses were the lack of neuroinflammatory findings in the CSF with a median cell count of 2 cells (IQR 1–5) and normal protein levels, median 0.463 g/L (IQR 0.286–0.622, all within normal limits when adjusted for age).

MRI was rarely normal and showed disease-specific patterns in 22/30 (73.4%) available descriptions. Most commonly, signs of neurodegenerative diseases (cerebellar atrophy, focal lobar atrophy, olivopontocerebellar degeneration) were present in 9/30 (30%) of cases along with direct evidence of primary or metastatic central nervous system tumors in 5/30 (16.7%) of cases. Other manifestations included acute cerebrovascular lesions and signs of infectious disease (viral encephalitis, Creutzfeldt-Jacob disease).

EEG findings were normal or non-specific in 18/30 (60%) available descriptions. Generalized slowing compatible with metabolic, infectious encephalopathy and neurodegenerative disorders was the most common abnormal pattern seen in 6/30 (20%) of cases whereas focal epilepsy restricted to the temporal lobe at examination was unique for limbic encephalitis and epilepsy cases. Differentials between cryptogenic epilepsy and autoimmune LE were MRI and CSF abnormalities, where in the former no signs of neuroinflammation were present.

### Antibody results

46 antibodies were detected from 39 serum but not CSF samples and anti-titin (N10), anti-recoverin (N11), anti-Yo (N8) anti-SOX-1 (N7) and anti- Amphiphysin (N7) comprised 43/46 (93.3%) of the cases and all were detected in serum. None were detected in CSF. In 36/45 (80%) of the cases lineblot band intensity was borderline/weak and only 1/45 (2.3%) of the cases of anti-titin had strong band intensity. However, in this case, limbic encephalitis with concomitant LGI-1 antibodies and typical faciobrachiodystonic seizures was diagnosed without evidence of cancer, malignant thymoma, signs of myasthenia gravis or additional clinical phenotypes. None of the cases with detectable antibodies had typical epidemiological associations with cancer or high-risk PNS phenotypes (4/8 (50%) anti-Yo in male patients without cerebellar syndrome). Detailed lineblot results are shown in Table 2.

**Table 2:** Lineblot band intensity in Cohort A.

Lineblot band intensity	Weak/borderline N (%)	Positive (+ / + +) N (%)	Strongly positive (+ + +) N (%)
N1 Anti-Hu	1 (100)	–	–
N0 Anti-Ri	–	–	–
N1 Anti-Ma1/2	1 (100)	–	–
N7 Anti-amphiphysin	6 (85.7)	1 (14.2)	–
N1 Anti-CV2/CRMP5	1 (100)	–	–
N8 Anti-Yo (PCA-1)	8 100)	–	–
Anti-Tr (DNER)	–	–	–
N10 Anti-titin	8 (80)	1 (10)	1 (10)
N11 Anti-recoverin	8 (72.7)	3 (26.2)	–
N7 Anti-SOX-1	4 (57.2)	3 (42.7)	–

*CRMP5* collapsin response-mediator protein-5, *DNER* Delta/Notch-like epidermal growth factor-related receptor, *PCA-1* Purkinje cell cytoplasmic antibody-1.

### Limbic encephalitis (LE) cases

**Figure 1:**
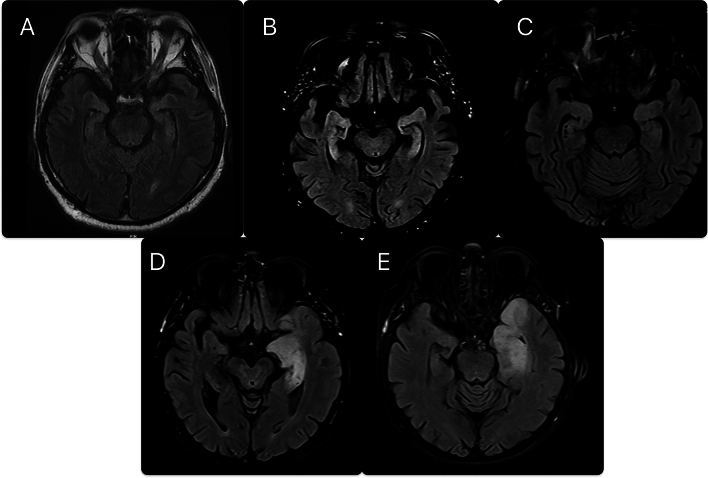
(**A**–**C**) from patients 1–3 respectively showing MRI FLAIR bilateral mesiotemporal lobe hypersignals;(**D**) Patient 5 initial MRI FLAIR sequence with left mesiotemporal hypersignal mistaken for autoimmune encephalitis; (**E**) Follow-up MRI of patient 5 showing expansion of the lesion beyond the limbic system and suggestive of neoplasm.

5/39 (12.8%) had either typical clinical or paraclinical finings compatible with LE and fulfilled criteria for possible AE. Further, 3/5 patients eventually fulfilled definite autoimmune LE criteria and were diagnosed accordingly, whereas 2/5 cases fulfilling only possible AE criteria were diagnosed with glioblastoma multiforme of the temporal lobe initially suspected as LE (patient 5, Table 3) and probable paraneoplastic LE (patient 4, Table 3, PNS-care score 7) for an unknown antibody due to close relation with non-small cell lung cancer and no evidence of metastatic central nervous system disease or alternative causes. MRI images are shown in Fig. 1.

**Table 3:** Limbic encephalitis and its mimics.

Patient number	Age	Gender	Concomitant conditions	Symptom duration, months	Chief complaint	MRI findings	EEG findings	Antibody detected	Response to immunotherapy	Additional information
1	67	Male	Hypertension	120	Anterograde amnesia, dementia	Bilateral mesiotemporal hypersignal	Focal temporal lobe epilepsy	Anti-recoverin	No improvement	–
2	78	Male	None	1	Anterograde amnesia	Bilateral mesiotemporal hypersignal	Focal temporal lobe epilepsy	Anti-CV-2, anti-recoverin	Improvement	–
3	80	Female	Hypertension	2	Anterograde amnesia, faciobrachiodystonic seizures	Subtle hypersignal in mesiotemporal lobes bilateraly	Focal temporal lobe epilepsy	Anti-LGI-1 Anti-amphiphysin Ant-titin	Improvement	–
4	70	Male	Squamous cell lung cancer	2	Anterograde amnesia, behavioral change, personality change, orientation difficulties and aggressiveness	Normal	Focal temporal lobe slowing	Anti-titin	No improvement	Inflammatory CSF: cells 22 Protein 0.664 G/L OCB + No carcinomatosis No infectious etiology
5	78	Female	None	1	Severe anterograde amnesia, personality change	Left mesiotemporal hypersignal;	Focal temporal lobe epilepsy	Anti-titin	Improved	Follow-up MRI reveals the expansion of hypersignal suggestive of tumor Biopsy confirms glioblastoma multiforme

*EEG* electroencephalography, *G/L* grams per liter, *LGI-1* leucine-rich glioma-inactivated protein 1, *MRI* magnetic resonance imaging.

All cases had antibodies in serum detected by lineblot (recoverin 2/5 and titin 3/5). 1/5 (20%) had concomitant LGI-1 autoimmunity while the rest were considered as false positive for titin and recoverin.

### Follow-up, evolution of disease and cancer associations

In patients with available follow-up > 12 months (20/39), during a median period of 27 months (IQR 17–33), none developed typical clinical syndromes or detectable cancers consistent with detected antibodies (myasthenia gravis, retinopathy, cerebellar syndrome respectively to three most detected antibodies—titin, recoverin and Yo). Disease evolution was heterogenous and followed initial diagnosis pattern: acute metabolic and neuroinfectious syndromes resolved whereas chronic neurodegenerative disorders followed a progressive pattern. PNS care score at the time of antibody detection was a median of 3 (IQR 1–3) and remained unchanged at end of follow-up (median 3 (IQR 1–3).

### Comparison between cohort A and cohort B

Definite AE/PNS cases (cohort A, N23) obtained from both commercial and in-house techniques were compared with cohort B (N35). For clarity, LE patients in Table 3 were excluded from this analysis because their antibody status is considered unknown as discussed previously.

General description of cohort A is shown in Table 4. Most common clinical phenotype was limbic encephalitis in 9/23 (39.1%) of cases presenting with short term memory loss in 7/9 (77.8%) and seizures in 5/9 (55.6%). All antibodies were diagnosed using commercial cell-based assays (CBA) with exceptions for KLHL-11, GFAP, GAD65 that were detected with rat brain immunohistochemistry. The later were detected in CSF exclusively.

**Table 4:** Descriptive and comparative data of cohort A and cohort B.

Definite AE /PNS patients			
N = 23			
General description			
Clinical phenotype N (%)			
Brainstem encephalitis 1 (4.3); Cerebellar syndrome 8 (34.8);Encephalomyelopoliradiculoneuritis 1 (4.3); Limbic encephalitis 9 (39.1); NMDARE 3 (5.1);Sensory neuronopathy 1 (4.3)			
Fulfils possible AE criteria N (%)			
11 (47.8)			
Fulfils definite LE criteria N (%)			
N11			
4 (36.4)			
Detected antibodies N (%)			
AMPAR 1 (4.3); CASPR2 3 (13); GABAB 1 (4.3); GAD65 2 (8.7); GFAP 1 (4.3);Hu 1 (4.3); KLHL-11 1 (4.3);LGI-1 3 (13); NMDA 3 (13;) Ri 1 (4.3); Yo 6 (26.1)			
Cancer detected N (%)			
SCLC 2 (20); Ovarian 7 (70); Testicular 1 (10)			

Comparative analysis			
	Definite AE /PNS patients	Non-AE patients	P value
	N=23	N=35	
Age, median (IQR)	58 (45–64)	65 (53-70)	0.283
Gender, female N (%)	16 (69.5)	20 (57.4)	0.413
Time from symptom onset to diagnosis, median months (IQR)	2 (1-7)	5 (1-17)	0.067
CSF pleocytosis N (%)	14 (60.8)	11 (31.4)	**0.042**
CSF cell count, median (IQR)	13 (5-62)	2 (1-5)	**0.007**
CSF proteinorachia N (%)	13 (68.4)	14 (60.9)	0.751
CSF protein count (g/L), median (IQR)	0.5 (0.37-0.75)	1 (14.3)	0.981
CSF OCB N (%)	**(N11) **7 (63.6)	N(7) 0 (0)	0.066
MRI features suggestive of encephalitis N (%)	6 (26.1)	0 (0)	**0.002**
EEG temporal lobe slowing/epilepsy N (%)	14 (60.9)	**(N30) **2 (6.7)	**0.0003**
Deceased, N (%)	8 (34.8)	4 (11.4)	**0.036**
PNS care score, median (IQR)	6 (4–10)	3 (1-3)	**0.002**
Number of samples tested positive on serum (%)	23/23 (100)	35/35 (100)	–
Number of positive serum samples retested positive on CSF (%)	10/23 (43.5)	–	–
Serum only tested, N (%)	13/23 (56.5)	35/35 (100)	–

*AE* autoimmune encephalitis, *AMPAR* α-amino-3-hydroxy-5-methyl-4-isoxazolepropionic acid receptor, *CASPR2* Contactin-associated protein-like 2, *CSF* cerebral spinal fluid, *EEG* electroencephalography, *GAD65* glutamic acid decarboxylase, *GABAB* gaba-aminobutyric acid B, *GFAP* glial fibrillary acicid protein, *KLHL-11* Kelch like protein- 11, *LE* limbic encephalitis, *LGI-1* leucine rich-glioma inactivated-1, *MRI* magnetic resonance imaging, *NMDAR* N-methyl-*D-aspartate* receptor, *OCB* oligoclonal bands; PNS- paraneoplastic neurological syndromes; SCLC- small cell lung cancer.

Significant values are in bold.

Discriminative features between cohort A and B were CSF pleocytosis (14/23 (60.8%) for cohort A vs. 11/35 (31.4%) for cohort B, p = 0.042, respectively), MRI features suggestive of encephalitis (6/23 (26.1%) for cohort A (5/6 (83.3%) mesial temporal lobe abnormalities) vs. 0 (0%) for cohort B, p = 0.002) and epilepsy restricted to temporal lobes (14/23 (60.9%) for cohort A vs. 2/30 (6.7%) for cohort B, p = 0.0003).

In cohort A, 11/23 (47.8%) fulfilled possible AE criteria and of those, 4/11 (36.4%) fulfilled definite LE criteria (regardless of antibody identified allowing definite diagnosis without satisfying all three major requirements of definite LE criteria). Reasons for non-compliance with the later criteria were chronic disease progression in 4/7 (57.1%) and lack of MRI abnormalities in 3/7 (42.9%) of the cases.

## Discussion

In the present study we report seroprevalence of various neuronal antibodies detected in serum using commercially available CBAs and lineblots in a spectrum of neurological and non-neurological disorders. Although most cases harboring neuronal antibodies had alternative causes for their underlying symptoms, a small subset of patients had clinical and paraclinical signs of AE. Altogether, this poses grounds for misdiagnosis of alternative causes of nervous system disorders as autoimmune due to similarities between non-autoimmune and autoimmune cases in demographical appearance and clinical presentations. To avoid misinterpretation of non-specific antibody results as significant and to avoid misdiagnosis of AE, differential elements should be discussed.

First, in contrast to previous epidemiological studies demonstrating higher incidence of neuronal surface antibody mediated AE rather than intracellular antibody mediated PNS^12,13^^,^ our data shows a high proportion of patients with antibodies against intracellular antigens detected by commercial lineblots. However, before establishing a diagnosis of AE or PNS, known caveats of commercial assays for the detection of intracellular antibodies need to be considered. Previous reports regarding Yo and SOX-1 amongst other antibodies suggest high rate of false positive results that are not confirmable using alternative antibody detection methods^6,7^. Our data adds to these findings demonstrating that anti-titin and anti-recoverin were most commonly detectable by lineblot in serum,however, in none of the cases were consistent with previously described paraneoplastic phenotypes^14,15^ and were found in miscellaneous neurological and non-neurological conditions. Moreover, for some antibodies, a positive correlation between definitive cases of PNS and lineblot band intensity has been established and might ease interpretation of commercial lineblot results^6^. It is in line with our findings where > 80% of detected antibodies by lineblot were borderline/weakly positive and had alternative diagnoses. On the other hand, we demonstrated one case of LGI-1 antibody-associated limbic encephalitis with typical faciobrachiodystonic seizures with concomitant strong (+ + +) anti-titin positivity on lineblot from serum. This patient improved after immunotherapy and never had signs of thymoma or myasthenia gravis. This in turn supports earlier recommendations that in most cases, results from lineblots alone without additional confirmation are not sufficient to establish the diagnosis^7,16^. Use of alternative CBA or rat brain immunohistochemistry techniques therefore might improve diagnostic accuracy^17^. However, confirmatory in-house antibody detection methods are currently restricted to a few immunological laboratories requiring specialized personnel for the preparation and interpretation of test results making this approach impractical in standard clinical settings. These diagnostic pitfalls of commercial tests are evident in our cohorts.

We described 3 patients with definite LE according to the criteria from Grauss et al^2^. All had either anti-titin or anti-recoverin antibodies present. Few reports suggest similar presentations of LE associated with the aforementioned antibodies^18–20^. However, there is currently no evidence of the expression of these proteins in the nervous system making the relationship between these antibodies and LE doubtful^21,22^. Moreover, as shown in the present series, unless compatible with phenotypes described previously^14,15^, titin and recoverin positivity on commercial lineblot should not be used as the markers of LE as they are present in various non-autoimmune neurological disorders. Reports suggesting associations between titin or recoverin antibodies and limbic encephalitis, may potentially mislead future researchers and clinicians adding to the growing problem of misdiagnosis of AE and PNS. The 3 cases described are circumstances when immunological laboratories should be consulted for the detection of additional antibodies in order to avoid misleading conclusions. However, given variable financial, socioeconomic and policy differences of national healthcare systems, collaboration with referral immunological centers may be inaccessible. Therefore, in clinical settings where extensive immunological testing is unavailable, strict adherence to the criteria for AE and PNS are necessary to ascertain the diagnosis.

The criteria for AE have been specifically designed to mitigate prompt initiation of immunotherapies in suspected cases without immunological verification reflecting common clinical scenarios where antibody testing is not available or takes weeks to perform^23^. Although the criteria for possible AE have lower specificity and may include mimics of AE as shown in previous publications^24^ as well as our cohort (case no. 5, Table 3), the criteria for probable and definite AE have been externally validated and show good concordance^4,24^. Moreover, for the diagnosis of PNS, updated criteria for PNS and the PNS-care score show improved specificity when compared to criteria published in 2004^25,26^. Therefore, with exceptions for brainstem encephalitis^4^ VGKC, IGLON-5, CASPR2, LGI-1 and rarely Ri antibody-mediated PNS known for their indolent clinical course and/or lack of neuroinflammatory features in the CSF^27–31^ , most immune mediated neurological syndromes have neuroinflammatory findings in CSF studies or neuroimaging and are mandatory findings in the criteria of AE to establish the diagnosis. Lack of neuroinflammatory features suggest alternatives causes as is evident from our cohorts where in most false positive cases, the clinical course is progressive rather than subacute and there was no evidence of neuroinflammatory features in paraclinical studies.

However, diagnosis of definite neurological autoimmunity may still be clinically challenging because current criteria of AE and PNS relies heavily on antibody detection and as discussed previously, due to major caveats in this aspect and in some cases low availability of antibody testing, a diagnosis of AE/PNS solely based on clinical grounds may be the only alternative for some centers. To ease clinical diagnosis a multistep approach using clinical scores should be considered. First, to differentiate between infectious and autoimmune aetiology in patients with signs of encephalitis, implementation of a risk score for autoimmune etiology, using simple and available analytes can be considered^32^. Second, when epilepsy of unknown cause is predominant, antibody prevalence in epilepsy (APE) may aid diagnosis of autoimmunity^33^. For brainstem predominant symptoms, the MATCH score designed to identify patients with KLHL-11 mediated syndromes is specific in reasonable clinical settings^34^. Finally, for cancer screening, when antibody status is not known, a full body CT may reveal signs of underlying tumours and aid in diagnosis^1^^,^ however, a recent study suggests a thoracic CT is the best starting point as small cell lung cancer related syndromes are most predominant^35^.

Importantly, our data is comparable in variable aspects to larger previously published studies.

First, careful consideration of alternative aetiologies is always required when differentiating MRI mesiotemporal lobe lesions, because it is one of the most common reasons for the misdiagnosis of AE to alternative aetiologies as shown in our cohorts and by Van Steenhoven et al.^24^.

Foremost, only a minority of samples positive for neuronal antibodies are reconfirmed in specialised laboratories if only the serum is tested. Our data indicate that 62% of serum samples may be false positive. It is line with Van Steenhoven et al. who reported that 56% were non-confirmable if retested. On one hand, this reflects the misuse of diagnostic tools for the detection of AE/PNS, because most of the patients are tested for AE/PNS without supporting evidence. On the other hand, even though no test is perfect, this shows that alternative diagnostic approaches are in desperate need.

It is currently unreasonable to suggest referral laboratories to do centralized neuronal antibody testing on an international scale, because the demand for neuronal antibody testing is much higher than the number of laboratories capable of testing. Moreover, although these institutions offer gold standard diagnostic methods, most are not certified as clinical laboratories creating legal issues for diagnostic purposes.

Therefore, the use of commercial assays is the current diagnostic standard. However, to improve diagnostic accuracy, validation of commercial tests using CSF samples is needed. Currently, some antibodies on commercial assays are validated to detect antibodies in serum despite the recommendations for CSF analysis from referral centers. Moreover, the number of antigens causative of AE is growing rapidly in research settings. However, the number of antigens on commercial panels has not been revised for years. CSF testing with the expansion of the number of antigens on commercial panels is needed to improve current clinical practice.

### Limitations

The major limitation of our study is the retrospective basis of alternative diagnoses only using clinical criteria with limited diagnostic assays available. Because in-house rat brain immunohistochemistry was mostly not available during the described study period, only a minority of patients were tested with both commercial and in-house diagnostic tests, while most had serum tested without CSF testing. However, it is possible that CSF testing on a number of serum positive lineblot samples were never sought due to the unlikeliness of an autoimmune central nervous system disorder and plausible alternative explanations of the patients’ symptoms. Counterwise, a proportion of serum samples positive for neuronal surface antibodies were never tested in CSF due to typical features of AE/PNS and cost-effectiveness. Also, because Lithuania does not have a referral laboratory for confirmatory neuronal antibody testing, it is possible that a minority of cases were misdiagnosed for alternative neurological disorders rather than AE. Another limiting factor from our as well as other studies is that for cost-effectiveness, negative samples on commercial CBAs and lineblots are almost never re-tested with in-house techniques. It is another possible reason for the underdiagnosis of AE/PNS. A small sample size and heterogeneity of the data are other limiting factors.

## Conclusions

The majority of antibodies against intracellular antigens detected by lineblots may produce non-specific results and may be found in patients with various non-autoimmune diseases of diverse etiologies. To differentiate between autoimmune and non-autoimmune etiologies, the adherence to clinical criteria, proof of neuroinflammation on paraclinical tests along with ancillary CSF testing should be sought. The use of in-house confirmatory techniques may improve diagnostic accuracy.

## Materials and methods

### Data acquisition

Health reports of patients who tested positive for at least one neuronal antibody using both commercially available and in-house diagnostic methods between 2016 and 2022 in Vilnius University Hospital Santaros Clinics were retrospectively evaluated. Clinicodemographic data, laboratory and neuroimaging testing results (Tables 1 and 4) were collected for patients included in this study.

### Patient selection

**Figure 2:**
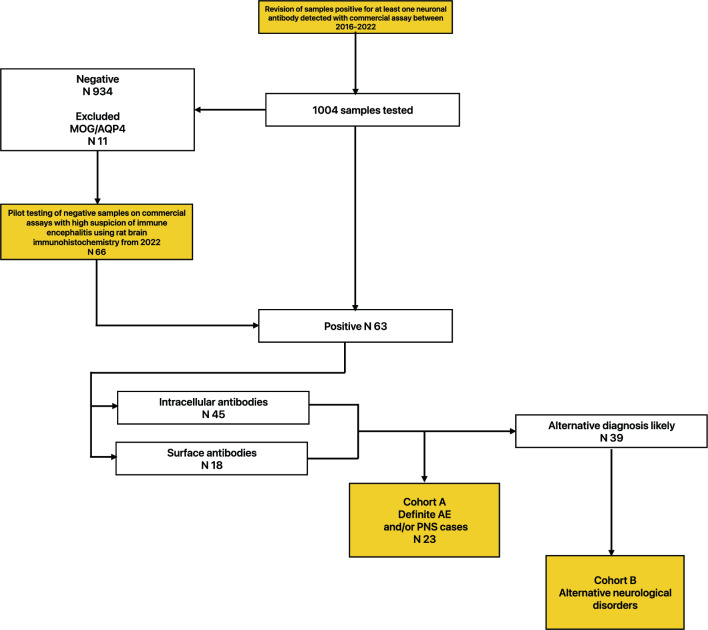
Flowchart depicting patient selection.

All patients positive for at least one neuronal antibody were included in the study and were retrospectively evaluated for the fulfilment of possible AE, probable, definite AE or PNS criteria when appropriate. Those with alternative diagnoses were included in the main cohort. Their clinical data was additionally compared with definite AE and PNS cases. A flowchart of the study is depicted in Fig. 2. Antibody-positive demyelinating disorders were not included in the study.

Additionally, anti-titin and anti-recoverin positive patients were evaluated for clinical and paraclinical signs of paraneoplastic malignant thymoma associated myasthenia gravis and paraneoplastic retinopathy described in original reports^14^^,^^15^ .

### Antibody detection

Neuronal antibody testing with commercial assays is currently available in three university hospitals across Lithuania. However, there is no referral center offering advanced confirmatory neuronal antibody testing. All hospitals mostly rely on commercial assays and clinical criteria to establish the diagnosis of central nervous system autoimmunity.

Commercially available indirect immunofluorescence cell-based assays (CBA) from Euroimmun, Lubeck, Germany, for the detection of neuronal surface antibodies (anti-N-methyl-D-Aspartate receptor (NDMAR), anti-leucine-rich glioma-inactivated protein 1 (LGI-1), anti-α-amino-3-hydroxy-5-methyl-4-isoxazolepropionic acid receptor (AMPAR), anti-contactin-associated protein 2 (CASPR2) and anti-gamma-amino-butyric acid B-receptor (GABAbR) were used as per manufacturer’s instructions. The samples were considered positive when the immunofluorescence was observed at the titers greater than 1:10. For commercial CBAs both serum and CSF were tested when available. Lineblots for intracellular antibodies against intracellular antigens (anti-Hu, Anti-Yo, anti-Ma1/2, anti-amphiphisin, anti-DNER, anti-CV2, anti-titin, anti-recoverin) from Euroimmun, Lubeck, Germany were used as per manufacturer’s instructions. For lineblots, only serum samples were tested.

Additionally, during the preparation of this manuscript (year 2023), frozen samples of seronegative patients on commercial assays described above, but with high clinical suspicion of AE/PNS were stored in Vilnius University Hospital Santaros Klinikos and tested at the Life Sciences Center of Vilnius University for additional neuronal antibodies using in-house indirect rat brain immunofluorescence assay. Briefly, ketamine and xylazine-sedated rats were perfused with isotonic sodium chloride and 4% paraformaldehyde. Removed half brains were post-fixed in 4% paraformaldehyde for 1 h, washed in phosphate buffered saline, placed in 30% sucrose for 24–48 h, and then frozen to -80 °C covered in Tissue Tek. Frozen brains were cut to 10–20 μm sagittal sections, rehydrated with phosphate-buffered saline for 10 min and blocked using 3% bovine serum albumin and 3% normal goat serum in phosphate-buffered saline for 1 h. Brain sections were then incubated with patient CSF or serum for 24 h at room temperature (dilution: CSF 1/10; serum 1/100). Slides were washed three times in phosphate-buffered saline and incubated for 1 h with Alexa Fluor 488-conjugated goat anti-human IgG (Thermo Fisher Scientific, Waltham, MA; dilution 1:500), and then with DAPI (1 µg/ml) for 10 min. After three rinses with phosphate-buffered saline for 15 min each, slides were mounted in Mowiol medium (Sigma-Aldrich, Saint-Louis, MO) and imaged under an Olympus CellVivo microscope system (Tokyo, Japan).

All positive samples obtained using rat brain immunofluorescence were reconfirmed in an outside reference laboratory (Lyon, France) using the same protocol as above.

## Data Availability

The datasets generated and/or analysed in the current study are available from the corresponding author upon reasonable request.
